# Glycaemic control and outcomes in children with type 2 diabetes diagnosed at or before 10 years of age

**DOI:** 10.1002/edm2.192

**Published:** 2020-10-20

**Authors:** Erin Greenup, Bhuvana Sunil, Mary Margaret Barr, Ambika P. Ashraf

**Affiliations:** ^1^ Department of Pediatrics/Division of Pediatric Endocrinology and Diabetes University of Alabama at Birmingham Birmingham AL USA; ^2^ University of Alabama School of Medicine University of Alabama at Birmingham Birmingham AL USA

**Keywords:** dyslipidaemia, paediatric, type 2 diabetes

## Abstract

**Background:**

Type 2 diabetes (T2DM) in children is considered rare before puberty.

**Objective:**

Describe the characteristics and outcomes of children with T2DM diagnosed at or before 10 years of age.

**Methods:**

Retrospective electronic medical record review of children diagnosed with T2DM at a University Children's Hospital over 12 years was conducted. Patient characteristics at diagnosis, 2‐3‐year follow‐up, and 4‐5‐year follow‐up were analysed as a whole and by age groups, 5‐8 and 9‐10 years.

**Results:**

There were 42 children ≤ 10 years with T2DM (5‐8‐year age group, n = 8 and 9‐10‐year age group, n = 34). There were 88.1% African American, 11.9% Caucasian, and 88.1% females. Body mass index (BMI) was ≥95th percentile in 95.2%. Average BMI z score was 2.5 ± 0.4 and higher in the 5‐8‐year age group (2.7 ± 0.5 vs 2.4 ± 0.4, *P* = .02). Average haemoglobin A1C at diagnosis was 10.5 ± 2.4%, and improvement was seen at 2‐3 years, but subsequent worsening was noted at 4‐5 years in both age groups. At 4‐5 years after diagnosis, 93.9% required insulin for management of their hyperglycaemia, 21.2% had hypertension requiring treatment, 28.6% had low‐density lipoprotein ≥130 mg/dL, and 28.6% had high‐density lipoprotein <40 mg/dL.

**Conclusions:**

T2DM at or below 10 years of age disproportionately affected females and ethnic minorities and was associated with morbid obesity. The majority of these children did not achieve glycaemic control and required insulin for management of their hyperglycaemia after 4‐5 years, indicating the need for increased awareness of T2DM and intensive treatment in this special group.

## Graphical abstract

Our study is a retrospective chart review of the glycaemic control and metabolic outcomes of 42 children diagnosed with type 2 diabetes (T2DM) at or below 10 years of age. It demonstrated the disproportionate disease burden of T2DM and aggressive disease progression to the need for insulin therapy for hyperglycaemia management amongst younger children with T2DM who are morbidly obese and at‐risk minorities. A better understanding of the epidemiology, treatment and outcomes is essential to achieve target goals and reduce complications.

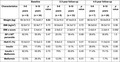

## 1 INTRODUCTION

Type 2 diabetes (T2DM) in children less than 10 years of age is rarely reported. Current American Diabetes Association (ADA) guidelines recommend consideration of risk‐based screening for prediabetes and/or T2DM in children after the onset of puberty or in those ≥10 years of age who are overweight or obese and have additional risk factors for diabetes.^1^ This may fail to recognize the onset of T2DM in younger children. There is paucity in our understanding of the trends in glycaemic control and complications of T2DM in this younger age group of children.

Little is known about the population of children diagnosed with T2DM prior to age 10. Studying this particular group is important because a large percentage of youth with T2DM may have comorbidities at baseline. This cohort is at risk for earlier development of complications and is expected to be exposed to these risk factors for a much longer period of time.^2^ It is estimated that in children diagnosed with diabetes at age 10 years, girls on average will lose 19 life‐years, and boys will lose 18.7 life‐years, with these numbers even higher in African Americans (AA).^3^

Recent estimates of the prevalence of obesity in children and adolescents show a continued upward trend with an overall prevalence of about 1 in 5 children.^4^ The prevalence of T2DM in children is estimated to be about 0.46 per 1000.^5^ Data from the SEARCH for Diabetes in Youth Study showed a relative increase in the prevalence of T2DM of 30.5% from 2001 to 2009 in those 10 to 19 years of age.^5^ The SEARCH database reported 11 children with T2DM under age 10 years in 2001^6^ and 19 children in 2009.^5^ A single‐centre study in South Texas reported 20 cases of T2DM diagnosed between 2000 and 2015 in children under age 10.^7^ A prospective national surveillance study in Canada identified 19 patients with newly diagnosed T2DM under age 10 over a 24 month period from 2006 to 2008.^8^ The Pediatric Diabetes Consortium (a group of eight paediatric diabetes treatment centres in the United States) identified 38 children under age 10 at diagnosis of T2DM, out of a total of 503 participants enrolled.^9^

The primary purpose of the proposed study is to describe the characteristics of children who were diagnosed with T2DM at or below 10 years of age at baseline and at follow‐up visits with focus on glycaemic control, blood pressure, and lipid measures. We also aimed to determine if there were any differences in the variables by age (ie between age groups 5‐8 years and 9‐10 years).

## 2 METHODS

**FIGURE 1:**
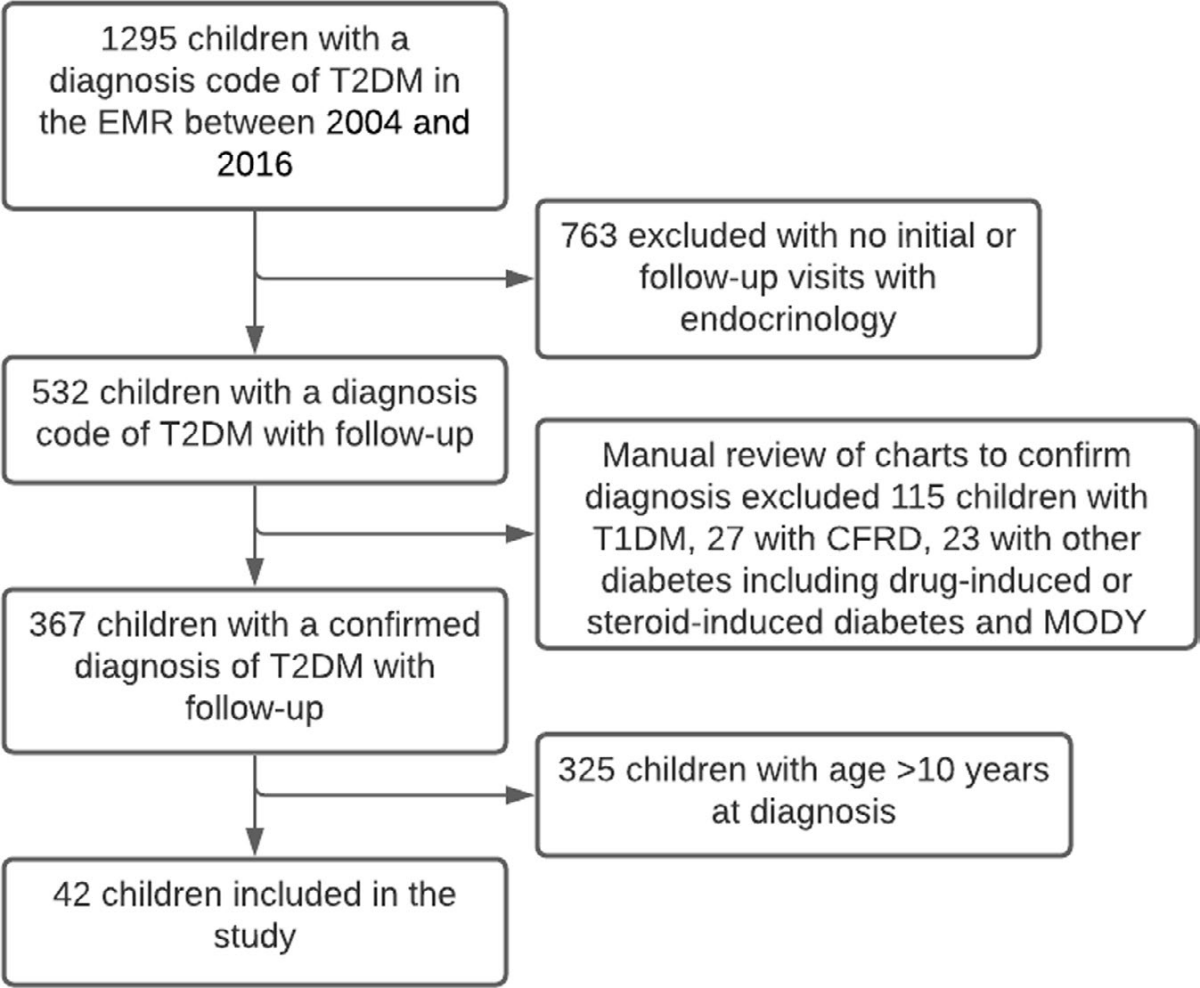
Flow chart representing patient inclusion/exclusion algorithm

This was a retrospective electronic medical record (EMR) review of paediatric patients diagnosed with T2DM between 2004 and 2016 by the Pediatric Endocrinology Division at the Children's Hospital of Alabama, University of Alabama at Birmingham (UAB). The research protocol was approved by the UAB Institutional Review Board for Human Use. The International Classification of Diseases (ICD‐9‐CM) diagnosis codes of 250.00 and 250.02 were used to identify all potentially eligible patients with T2DM. Investigator EG manually reviewed the medical records of subjects included in the study and reviewed the longitudinal data to ascertain that the patients had T2DM. Inclusion criteria were age 10 years or younger at the time of diagnosis, haemoglobin A1C (HbA1C) ≥6.5%, and a diagnosis of T2DM based on the endocrinologist's assessment. Exclusion criteria included diagnoses of type 1 diabetes, maturity onset diabetes of the young, cystic fibrosis‐related diabetes, chronic renal or pancreatic disease, Prader‐Willi syndrome, or conditions requiring chronic systemic steroid use or immunosuppression and patients who did not have more than one visit with the endocrinologist. Figure 1 represents a flow chart for the final patients included for analysis in the study. Data were abstracted from initial diagnosis, and follow‐up information was collected at 2‐3 years and 4‐5 years after diagnosis when available. If a patient had available parameters for more than one visit at the time interval, the visit closest to 2 years and 5 years from the diagnosis date was used. When a visit was not available right at the 2 year or 5 year mark, the next available visit between 2 to 3 and 4 to 5 year window was used.

Race and ethnicity were determined based on parental reports documented in the EMR. ‘Optimal glycaemic control’ was defined as achieving HbA1C < 7% based on the treatment target goal as defined by the ADA Standards of Care 2020.^10^ ‘Durable glycaemic control’ was defined as HbA1C < 8% as classified in the TODAY trial.^11^

All children with T2DM received similar diabetes and nutritional education, according to the UAB Endocrinology Division protocol and patients were given the same instructions to contact their paediatric endocrinologist frequently for medication adjustments to maintain euglycaemia. Medication and insulin treatment were initiated depending on HbA1C levels and at the discretion of the attending endocrinologist. Insulin therapy included long‐acting with or without rapid‐acting insulin preparations. Insulin doses were adjusted upon review of current blood sugar readings based on the division's protocol.

The study subjects were divided into two age groups by age at diagnosis, 5‐8 years and 9‐10 years, assuming the older age group of children was peripubertal. Documentation of Tanner staging was not always available due to the retrospective nature of the study. Hypertension was defined as blood pressure (BP) ≥95th percentile based on age, sex, and height percentile.^12^ Microalbuminuria was defined as an elevated urine albumin‐to‐creatinine ratio of >30 mg/g creatinine.^10^

### 2.1 Statistical analysis

All statistics were analysed by SAS v 9.4. For continuous variables, means and standard deviations were calculated by using the Wilcoxon test to compare the difference of the older and younger age groups at baseline, 2‐3‐year follow‐up, and 4‐5‐year follow‐up visits. The Fisher exact test was used to detect the proportional differences of the two age groups at these three predetermined time points of follow‐up.

## 3 RESULTS

A total of 42 children with T2DM diagnosed at ≤10 years of age were included in the study. There were 8 children diagnosed at 5‐8 years of age and 34 children diagnosed at 9‐10 years of age. In all of the 8 children in the younger group, the diagnosis of T2DM was made when HbA1C levels were checked due to symptoms of diabetes. All of them had polyuria, polydipsia or polyphagia, four children also had a diagnosis of vaginal candidiasis and 1 child also had reported weight loss.

In the older group, the diagnosis of T2DM was made when HbA1C was checked because of symptoms including polyuria, polydipsia or polyphagia (24 of 34, 70.6%), weight loss (4 of 34, 11.8%) and/or vaginal candidiasis (3 out of 34, 8.8%). Eight children (23.5%) did not have any symptoms of T2DM and were screened at a routine visit with their paediatrician due to coexistent obesity.

A retrospective analysis of growth records prior to the onset of diabetes available in 38 children revealed that 15.8% were low birth weight (LBW), that is <2500 g, and 13.2% were high birth weight (HBW), that is >4000 g.^13^ Of the 7 children in the younger age group, one (14.3%) had LBW and one (14.3%) had HBW. Of the 31 in the older group, 5 (16.3%) had LBW and 4 (12.9%) had HBW. Four out of 38 children in our study cohort were born prematurely (<37 weeks gestational age), 2 (28.6%) in the younger group and 2 (6.5%) in the older group.

### 3.1 Anthropometric measures and trends

The baseline characteristics of the patients are illustrated in Table 1. The majority were female, 87.5% females in the 5‐8‐year group and 88.2% in the 9‐10‐year group. Overall, the study population was 88.1% AA and 11.9% Caucasian. BMI was ≥95th percentile in 95.2% and ≥85th percentile in 100% of our cohort. In the younger group, 50% of patients had two parents and/or siblings with T2DM and all but one had a first‐degree relative with T2DM. In the 5‐8‐year age group, 87.5% had a parent with diabetes compared to 67.6% in the 9‐10‐year age group. All patients had reported family history of diabetes in their extended family.

**TABLE 1:** Baseline demographic and clinical characteristics of children diagnosed with type 2 diabetes at or before 10 y of age

Characteristics	Overall (n = 42)	5‐8‐y group (n = 8)	9‐10‐y group (n = 34)
Female sex	37 (88.1%)	7 (87.5%)	30 (88.2%)
Race			
African American	37 (88.1%)	6 (75%)	31(91.2%)
Caucasian	5 (11.9%)	2 (25%)	3 (8.8%)
BMI ≥ 99th percentile	33 (78.6%)	7 (87.5%)	26 (76.5%)
BMI ≥ 95th percentile	40 (95.2%)	7 (87.5%)	33 (97.1%)
Acanthosis nigricans	40 (95.2%)	8 (100%)	32 (94.1%)
Parent/sibling with T2DM			
Two parents or siblings	11 (26.2%)	4 (50%)	7 (20.6%)
One parent or sibling	20 (47.6%)	3 (37.5%)	17 (50%)
None	11 (26.2%)	1 (12.5%)	10 (29.4%)

Note

Values expressed as number of patients (percentage).

Abbreviations: BMI: body mass index, T2DM: type 2 diabetes.

The anthropometric data at baseline and subsequent follow‐up are depicted in Table 2. The average initial BMI z score was 2.5 ± 0.4. The BMI z scores were statistically different between the two age groups (2.7 ± 0.5 in the younger group vs 2.4 ± 0.4 in the older group, *P* = .02) at diagnosis, but not different at follow‐up visits.

**TABLE 2:** Characteristics of the patients at diagnosis and follow‐up

Characteristics	Baseline			2‐3‐y follow‐up			4‐5‐y follow‐up		
	5‐8 y (n = 8)	9‐10 y (n = 34)	*P* value	5‐8 y (n = 8)	9‐10 y (n = 33)	*P* value	5‐8 y (n = 7)	9‐10 y (n = 26)	*P* value
Weight (kg)	59.3 ± 14.0	75.3 ± 20.7	**.03**	72.2 ± 15.0	87.6 ± 23.8	.07	88.0 ± 12.7	93.6 ± 25.6	.76
BMI (kg/m^2^)	32.6 ± 7.0	32.9 ± 7.7	.73	32.6 ± 4.6	34.3 ± 7.5	.72	36.0 ± 5.6	35.5 ± 7.9	.56
BMI *z* score	2.7 ± 0.5	2.4 ± 0.4	**.02**	2.5 ± 0.3	2.3 ± 0.4	.15	2.4 ± 0.4	2.2 ± 0.4	.09
BP ≥ 95th percentile	62.5%	55.9%	.47	25.0%	39.4%	.69	28.6%	30.8%	1.0
HbA1C (%)	10.4 ± 3.0	10.5 ± 2.3	.99	9.9 ± 3.2	8.8 ± 2.6	.41	10.2 ± 3.4	11.0 ± 2.6	.60
Insulin	25%	17.6%	.63	12.5%	9.1%	.77	28.6%	19.2%	.59
Insulin + Metformin	62.5%	55.9%	.73	75%	60.6%	.45	71.4%	73.1%	.93
Metformin	12.5%	26.5%	.48	12.5%	30.3%	.31	0%	7.7%	.45

Note

Values expressed as mean ± standard deviation or percentage. The bold values indicate statistical significance.

Abbreviations: BMI, body mass index; BP, blood pressure; HbA1C, haemoglobin A1C.

Baseline C‐peptide levels were obtained in 35 patients. The average C‐peptide levels were not different between the two age groups: 4.77 ± 2.59 ng/mL (n = 7) in the 5‐8‐year age group vs 3.22 ± 2.36 ng/mL (n = 28) in the 9‐10‐year age group, *P* = .14.

Of the 42 patients, 39 had pancreatic autoantibodies obtained at diagnosis. All except four patients (10.3%) had negative autoantibody titres. In those with negative autoantibodies, two patients (9‐10 years old) presented with diabetic ketoacidosis (DKA). None of the patients presented with the hyperosmolar hyperglycaemic state. Of the four patients with positive GAD65 antibodies, the titres ranged from 0.5 to 2.8 U/mL (positive ≥ 0.5 U/mL). Two of these patients had elevated serum insulin levels at diagnosis (51.3 and 56.2 uIU/mL) prior to initiation of treatment consistent with β‐cell reserve, and one of them was managed with metformin monotherapy for 3 years prior to requiring insulin treatment, further corroborating our diagnosis of T2DM. All of the antibody positive patients were obese with acanthosis, and did not present with any episodes of DKA despite reported issues with medication adherence and elevated HbA1C ranging from 9% to >14%.

### 3.2 Glycaemic control and management

Table 2 illustrates longitudinal follow‐up data of HbA1C and treatment prescribed. The overall average HbA1C at diagnosis was 10.5 ± 2.4% and was not different between the two age groups. 41 patients had follow‐up data available at 2‐3 years after diagnosis, of which 29.3% patients achieved optimal glycaemic control and 41.4% patients achieved durable glycaemic control. Overall average HbA1C was 9.0 ± 2.7% and the HbA1C was not different between the two age groups. Follow‐up data were available in 33 patients at 4‐5 years after diagnosis, of which 12.1% patients achieved optimal glycaemic control and 18.2% achieved durable glycaemic control. Average HbA1C at 4‐5 years of follow‐up was 10.8 ± 2.7% and not statistically different between the age groups. Overall, a mild improvement in HbA1C was observed at 2‐3 years, but subsequent worsening was noted at 4‐5 years in both age groups, as illustrated in Table 2.

At diagnosis of T2D, 62.5% of patients in the younger group were treated with insulin and metformin, 25% were treated with insulin only, and 12.5% were treated with metformin only. In the older group, 55.9% were treated with insulin and metformin, 17.6% were treated with insulin only, and 26.5% were treated with metformin only.

At the 2‐3‐year follow‐up, in the younger group, 75% were treated with insulin and metformin, 12.5% with insulin only, and 12.5% with metformin only. In the older group, 60.6% were treated with insulin and metformin, 9.1% with insulin only, and 30.3% with metformin only. In the 9‐10‐year age group, two patients were also treated with glyburide and one patient was treated with liraglutide. At the 4‐5‐year follow‐up, in the younger group, 71.4% were treated with insulin and metformin, 28.6% with insulin only, and none with metformin only. In the older group, 73.1% were treated with insulin and metformin, 19.2% with insulin only, and 7.7% with metformin only. In the older group, one patient was also treated with pioglitazone. Overall, at the 2‐3‐year follow‐up, 73.2% of children were on insulin and at the 4‐5‐year follow‐up 93.9% were on insulin.

### 3.3 Blood pressure and microalbuminuria

At baseline, 61.5% of patients had BP documented in the hypertensive range. The younger group had a higher prevalence of 62.5% compared to 55.9% in the older group (Table 2). For the entire cohort, at the 2‐3‐year follow‐up, 36.6% of patients had BP in the hypertensive range and 14.6% were on antihypertensive medications. At the 4‐5‐year follow‐up, 30.3% of patients had BP in the hypertensive range and 21.2% of the patients were on antihypertensive medications.

Urine microalbumin was obtained in 5 patients at diagnosis, and was elevated in 2 of these patients. At the 2‐3‐year follow‐up, urine microalbumin was evaluated in 26 patients and elevated in 3 patients, and at the 4‐5‐year follow‐up, it was obtained in 24 patients and elevated in 4 patients.

### 3.4 Dyslipidaemia

Of the total 42 subjects, 27 had a lipid panel performed at the time of diagnosis. Total cholesterol (TC), low‐density lipoprotein cholesterol (LDL), high‐density lipoprotein (HDL), and non‐HDL were similar between the two age groups (Table 3). Serum non‐fasting triglycerides were higher in the younger group compared to the older group (271 ± 101 mg/dL vs 149 ± 75.2 mg/dL, *P* = .02). At diagnosis, LDL was ≥130 mg/dL in 25.9% of patients and HDL was <40 mg/dL in 51.9% of patients. Non‐HDL was ≥145 mg/dL in 48.2% of patients.

**TABLE 3:** Lipid measurements at diagnosis and follow‐up

Lipid value (mg/dL)	Baseline			2‐3‐y follow‐up			4‐5‐y follow‐up		
	5‐8 y (n = 5)	9‐10 y (n = 22)	*P* value	5‐8 y (n = 6)	9‐10 y (n = 28)	*P* value	5‐8 y (n = 6)	9‐10 y (n = 22)	*P* value
TC	180 ± 26.5	171 ± 27.0	.48	170 ± 18.3	154 ± 31.4	.17	180 ± 58.5	182 ± 38.8	.70
LDL	102 ± 31.0	107 ± 29.0	.71	99.5 ± 30.8	94.3 ± 29.6	.58	112 ± 57.7	116 ± 34.3	.62
HDL	35.6 ± 10.4	38.8 ± 9.1	.54	44.2 ± 14.4	43.8 ± 9.3	.75	55.5 ± 21.7	44.8 ± 10.7	.32
Non‐HDL	145 ± 31.3	131 ± 29.0	.50	126 ± 30.9	110 ± 31.3	.18	125 ± 60.8	137 ± 39.9	.42
TG	271 ± 101	149 ± 75.2	**.02**	133 ± 55.4	130 ± 74.5	.65	111 ± 36.8	188 ± 121	.08

Note

Values expressed as mean ± standard deviation. The bold values indicate statistical significance.

Abbreviations: HDL, high‐density lipoprotein; LDL, low‐density lipoprotein; TC, Total cholesterol; TG, triglycerides.

At the 2‐3‐year follow‐up, lipid profile data were available for 34 patients. The lipid values were not different between the two age groups. LDL was ≥130 mg/dL in 11.8%, HDL was <40 mg/dL in 38.2%, and non‐HDL was ≥145 mg/dL in 11.8% of patients. At the 2‐3‐year follow‐up, 5.9% of the patients were on a lipid‐lowering medication.

At the 4‐5‐year follow‐up, lipid measurements were available for 28 patients and similar between the two age groups. LDL was ≥130 mg/dL in 28.6%, HDL was <40 mg/dL in 28.6%, and non‐HDL was ≥145 mg/dL in 35.7% of patients. At the 4‐5‐year follow‐up, 7.1% of the patients were on a lipid‐lowering medication.

## 4 DISCUSSION

To our knowledge, this is the largest single‐centre cohort of paediatric T2DM in children ≤10 years of age and provides vital information regarding outcomes in this group. We found that there is rapid worsening of the disease leading to requirement of insulin therapy, poorer glycaemic control, and adverse lipid measures at follow‐up in younger children with T2DM. In our study, T2DM diagnosed at or below age 10 was predominantly seen in females, AAs, and mostly in children with BMI > 99th percentile, which is similar to data reported in older children and adolescents.^14^, ^15^, ^16^

All of the patients in our study were diagnosed with T2DM by a paediatric endocrinologist based on clinical, anthropometric, and laboratory data, as well as longitudinal follow‐up course. 10.3% had positive GAD65 antibodies, which is equal to the rate of antibody positivity in clinically diagnosed T2DM in the TODAY study,^16^ and lower than the 21.2% reported in the SEARCH study.^17^

The majority of our study population were AA and we had a significant female preponderance (88.1%), compared to the TODAY study (64.9%).^16^ Our data reflect the significant burden of T2DM on ethnic minorities. The most recent composition of Alabama's paediatric population was 29.3% AA, 7.7% Hispanic, and 57.8% Caucasian.^18^ As the only free‐standing children's hospital in the state of Alabama, this distribution represents our overall referral base. Of the children with T2DM who are seen in the paediatric diabetes clinic at our institution, the majority are AA (76%) and female (70%). ^19^ In patients diagnosed between 2004 and 2016 with established follow‐up with our hospital, 11.4% of the children were 10 years of age or younger.

It has been long‐established that birth weight has a U‐shaped relationship with development of adult‐onset T2DM, with both LBW and HBW being risk factors.^20^, ^21^ In our population, we were able to identify that a sizeable proportion of the study cohort was either premature, LBW or HBW. This observation may be attributable to the thrifty phenotype of these babies, with early foetal programming towards an insulin‐resistant milieu.^21^

We found that younger children (5‐8‐year age group) had a higher initial BMI z score and had parent(s) and/or sibling(s) with T2DM. A strong genetic predisposition is a crucial determinant for developing T2DM. Factors that trigger the phenotypic expression of the T2DM are the rapidity and degree of weight gain, consumption of high‐calorie food, physical inactivity, and certain diabetes‐inducing medications, which are additive to genetic susceptibility. This gene‐environment interaction may be responsible for younger age of onset and rapid progression.^22^

Glycaemic control declined rapidly over 4‐5 years in our population with nearly all of the children requiring insulin therapy at the 4‐5‐year follow‐up. This is significantly higher than the 46% of patients 10‐17 years of age from the TODAY study with treatment failure (persistently elevated HbA1C or need for insulin therapy),^23^ highlighting a likely more progressive phenotype of β‐cell decline and barriers to treatment with socio‐economic disparities. Compared to the TODAY study involving children and adolescents with T2DM of all age groups at 4 year follow‐up, our population had a higher average BMI z score and a higher percentage of children with LDL ≥ 130 mg/dL (10.75% vs 28.6%) at 4‐5‐year follow‐up.^23^

In the SEARCH study looking at youth diagnosed with T2DM age <20, the prevalence of diabetic kidney disease was 20% and the prevalence of hypertension just over 20% at age 21.^24^ In the TODAY cohort, youth age 10‐17 years at diagnosis of T2DM, after 3.9 years 16.6% had microalbuminuria and 33.8% had hypertension.^23^ In our population of young children, 16.7% had microalbuminuria and 21.2% had hypertension requiring treatment at the 4‐5‐year follow‐up, similar both SEARCH and TODAY follow‐up data. Clinicians need to be aware that comorbidities start early in the disease process in young children with T2DM.

The strengths of this study include unusually early presentation of T2DM and the long duration of follow‐up for the majority of our cohort compared to other paediatric T2DM studies that include mostly adolescent patients.^25^ Our study was inclusive of a large number of young AA patients in whom there is paucity of data on the natural history of T2DM.

Limitations of this study include the retrospective nature and that the data were collected from a single centre, and therefore, the study findings may not be generalizable to other populations. Due to the retrospective nature of the study, there were missing pubertal staging, C‐peptide and autoantibody data in a small number of patients. It is unclear if earlier pubertal onset and oestrogen exposure had contributed to the occurrence of diabetes at a younger age. The retrospective nature of the study precluded us from clarifying whether the lipid profile, especially the triglyceride levels, was obtained fasting. There may also be a selection bias in the follow‐up data as those patients who achieved remission or were well‐controlled on metformin monotherapy may have followed up with their paediatricians alone. We were also unable to assess adherence to treatment. While the intended target HbA1C per the ADA during the period of treatment from 2004‐2016 was higher (<8%), a target of <7% was used for data analysis based on the standard of care at the time of analysis per the 2020 ADA Standards of Care and the International Society for Pediatric and Adolescent Diabetes definition of optimal glycaemic control.

## 5 CONCLUSIONS

Our study demonstrates the disproportionate disease burden of T2DM and aggressive disease progression with continued requirement of insulin therapy amongst younger children with T2D who are morbidly obese and at‐risk minorities. A better understanding of the epidemiology, treatment and outcomes is essential to achieve target goals and reduce complications.

## CONFLICTS OF INTEREST

The authors declare no conflicts of interest.

## AUTHOR CONTRIBUTION

MMB and AA designed the research study. EG, MMB and BS collected the data. EG, MMB, BS and AA performed the research. EG wrote the first draft. All authors analysed the results and prepared the manuscript. All authors approved the final version of the manuscript.

## Data Availability

The data that support the findings of this study are available from the corresponding author upon reasonable request.
